# Diverse flower-visiting responses among pollinators to multiple weather variables in buckwheat pollination

**DOI:** 10.1038/s41598-023-29977-z

**Published:** 2023-02-22

**Authors:** Tadashi Miyashita, Shouta Hayashi, Kae Natsume, Hisatomo Taki

**Affiliations:** 1 Laboratory of Biodiversity Science, Faculty of Agriculture and Life Sciences, The University of Tokyo, Bunkyo-ku, Tokyo, 113-0032 Japan; 2 Forestry and Forest Products Research Institute, 1 Matsunosato, Tsukuba, Ibaraki 305-8687 Japan

**Keywords:** Ecology, Ecology

## Abstract

Response diversity to environmental change among species is important for the maintenance of ecosystem services, but response diversity to changes in multiple environmental parameters is largely unexplored. Here, we examined how insect visitations to buckwheat flowers differ among species groups in response to changes in multiple weather variables and landscape structures. We found differences in responses to changes in weather conditions among insect taxonomic groups visiting buckwheat flowers. Beetles, butterflies, and wasps were more active in sunny and/or high-temperature conditions, whereas ants and non-syrphid flies showed the opposite pattern. When looking closely, the different response pattern among insect groups was itself shown to be different from one weather variable to another. For instance, large insects were responsive to temperatures more than small insects while smaller insects were responsive to sunshine duration more than large insects. Furthermore, responses to weather conditions differed between large and small insects, which agreed with the expectation that optimal temperature for insect activity depends on body size. Responses to spatial variables also differed; large insects were more abundant in fields with surrounding forests and mosaic habitats, whereas small insects were not. We suggest that response diversity at multiple spatial and temporal niche dimensions should be a focus of future studies of the biodiversity–ecosystem service relationships.

## Introduction

Global environmental changes have led to concern about the deterioration of ecosystem functions and ecosystem services^1,2^. The conservation of biodiversity may mitigate these effects to some degree, which is a central argument of the biodiversity–ecosystem functioning debate. One of the main rationales is that differences in responses to environmental changes among species provide a basis for preventing the deterioration of ecosystem functions and services through niche complementary^3,4^ (i.e., community members with different niches complementarily cover a wide range of environmental niche space). Differential responses against environmental change among species in a community are termed “response diversity” and are expected to stabilize the ecosystem functions against disturbance^5^. Response diversity was applied originally to the compensatory population dynamics of different organisms in response to environmental change, but later came to embrace behavioral responses, such as pollinator visitations in response to weather conditions^6–12^, predator activity to air temperatures^13^ and herbivory rate in response to food quality^14^. Although the conceptual notion that response diversity plays a significant role in ecosystem service stability is now widely recognized, the extent to which response diversity contributes to the stability and enhancement of ecosystem services in the real world is not clear^15,16^.

The relationship between diversity and ecosystem services is particularly well-studied for pollination, both locally and globally^17–19^. This is not only because global food production and human health are highly dependent on pollination services, with 75% of crops requiring animal pollination^20,21^, but also because diverse pollinators potentially sustain crop pollination services. Pollination services are currently facing major threats, including landscape change and climatic change^22–25^. A number of studies have revealed that heterogeneity in landscape structure and the existence of natural land increase the abundance and species richness of pollinators and maintain crop production^6,26,27^. However, responses to land use change vary among species and taxonomic groups^16,28,29^. Syrphid flies may partly compensate for decreases in bees and butterflies under land use intensification^10,30^. This variation (i.e., response diversity) is thus likely to stabilize pollination services under landscape change.

Climatic change may also affect pollination services, not only through the decline of pollinator abundance and richness^25,31^) but also via changes in pollinator activity as well as phenological mismatches between pollinators and crop plants^22,32,33^. However, as pollinator responses to changes in weather are diverse^6,34,35^, complementary responses could mitigate the detrimental effects of climate change. For instance, honeybees are active at high temperatures on sunny days, whereas some native bees and hoverflies are active in cloudy and low-temperature conditions^36,37^, indicating the temporal complementation from response diversity of pollination services. However, there are still few examples of the simultaneous evaluation of the temporal and spatial complementarity of pollinators at the landscape level^38–40^. Furthermore, most studies of pollination services have focused on bees and hoverflies, with relatively few comprehensive analyses of visitors belonging to various taxa^38,41^. Considering that extreme weather events that may affect pollination services have recently increased in frequency^25,42^, clarification of spatiotemporal complementarity among diverse insects is an urgent task.

Here, we investigated how insect visitation to buckwheat flowers responds to spatiotemporal environmental change. Buckwheat is a self-incompatible plant with distylous flowers (long- and short-styled flowers), and benefits strongly from insect pollination^43^. Demand for buckwheat is increasing owing to the growing interest in health foods that include rutin and flavonoids^44^. Various insect taxa with a range of body sizes visit the flowers^45^, and non-bee small insects may contribute to seed production^43^. Therefore, buckwheat is well suited for the investigation of the responses of various flower visitors to spatiotemporal environmental changes and may provide empirical evidence for general issues related to response diversity and ecosystem services.

Our research consisted of two steps, namely, identifying insect response diversity to environmental changes and investigating the relationship between insect visitation and the seed set of buckwheat. The first step was aimed at determining whether different insect taxa exhibit different responses to changes in weather conditions (”behavioral” response diversity) and spatial structures of land use (“behavioral/population” response diversity) with regard to flower visitations. In particular, we hypothesized that large insects (> 10 mm body length) will increase in activity with increasing temperature and sunshine duration, while small insects decrease in activity with increasing temperature and sunshine duration, contributing to weather-dependent complementarity between groups. This expectation is based on the crude positive association between body size and thermal niches of flower-visiting insects that include a broad taxonomic range; lighter insects generally prefer lower temperatures due to increased water loss derived from an increased surface/volume ratio^10,46^. Although there are species- or taxon-specific differences in preferable weather conditions^7–9,12,39,47,48^, a trait-based approach across a broad taxonomic range, which is often used for ecosystem service assessment^49,50^, may provide insight into the resilience of pollination services under climate change. Similarly, the responses to land cover are likely to differ among insects with different sizes, given that the relative abundance of large insects is known to decrease with land-use intensity^10,25,51^. In the second step, we evaluate whether the number of visiting insects is translated into pollination services. We separately evaluated the contributions of large insects and small insects to the seed set, as in the first survey. For this evaluation, the seed set in the control group (open pollination with unlimited access to flowers for all insects) was compared with that when inflorescences were covered with a bag, through which small insects (< 10 mm body length) could pass but large insects could not.

We evaluated the hypotheses that (1) large insects increase in visitation frequency with increasing temperature and sunshine duration, while small insects decrease in visitation frequency in such conditions, (2) the seed set increases with the increasing visitation by insects, and (3) the relative contributions of large insects to seed set increases with increasing area of surrounding semi-natural land use.

## Materials and methods

### Study area

We conducted field surveys in the town of Iijima, Nagano Prefecture in central Japan (35°38′ to 35°43′ N, 13°53′ to 137°57′ E). The average annual precipitation is 1421.9 mm and the annual average temperature is 11.3 °C. Iijima is located in the narrow basin formed along the west side of the Tenryu River at an altitude of 600–700 m. The landscape has a fine-scale mosaic structure including farmlands (mostly rice paddy fields and buckwheat fields), residential areas, and fragmented forests (mixed with conifer plantations and broadleaved trees). Common buckwheat is cultivated twice a year: summer (May–July) and autumn (August–October). Cultivation and harvest are managed with the same schedule by a local agricultural association. The buckwheat fields are located on a flat terrace in an area of approximately 5 km × 5 km. In total, 14 buckwheat fields were selected as study sites with sizes ranging from 841 to 2419 m^2^ (average 1866 m^2^) (see approximate locations of buckwheat fields in Supplementary Fig. 2). The distance between adjacent sites ranged from 260 to 1170 m. All surveys and experiments were performed in the autumn of 2019.

### Insect observation

We observed visiting insects at the 14 study sites on all 11 days between September 8th and 22nd. The same observers visited all study sites (buckwheat fields) from 9 a.m. to 2 p.m. on 11 different non-rainy days. The order of sites visited within a day was changed daily, so as not to bias the time of day each site was visited. At each site, a 0.5 m × 5 m quadrat was established along each of the four edges of a rectangular buckwheat field, and observers recorded the number of insect individuals that landed on flowers in the quadrats for 12 min in total (3 min per quadrat). To prevent data bias due to differences in catching skills and handling time by the observers, the insects were not caught. Since we followed flying insects at a quadrat, there must have been only a little, if any, double counting of individuals. Some insect records were not precise (for instance, non-syrphid Brachycera species (order Diptera) were recorded as “non-syrphid flies” and Syrphid dipteran species were recorded as “hoverfly”). Other Dipteran species were recorded in as much detail as possible. Ants and beetles were identified to the species level whenever possible. Bees, butterflies, and wasps were identified to the family level. Other insects, such as stink bugs and grasshoppers, were recorded in as much detail as possible. Body sizes of insects were categorized as small and large with the threshold of 4 mm body width, with a visual inspection. There were two reasons for dividing insects into these two size classes. First, this size threshold corresponds to the mesh size of bags used for covering inflorescences in the experiment; small insects would be able to pass thorough the mesh while large insects would not (see “Pollination experiment”). Second, this size criterion corresponds approximately to the middle of the body mass range of insects (about 10 mg) reported in an earlier study^22^ that examined the allometric relation between the body mass of flower-visiting insects and thermal niches (see Supplementary Fig. S1 for the relationship between body width and body mass of major insects in our study area). As a result of the classification, large insects consisted of about 38% of the total insects while the rest was small insects (see “Results” for details).

We obtained weather data for the AMeDAS site (35°N, 138°E) in Iijima from the database of the Japan Meteorological Agency^52^. It was assumed that insect visitations were affected by short-term weather conditions immediately before the observation. Hence, the mean sunshine duration, temperature, and wind speed were calculated for the 30-min period before each observation. Humidity was not used as a variable owing to its strong correlation with time of day (*r* =  − 0.646).

### Pollination experiment

We bagged inflorescences to evaluate the separate contributions of small and large insects to buckwheat pollination. The bags (approximately 10 × 20 cm) were made of clear-colored polyethylene, with a mesh size of 4.5 × 4.5 mm, allowing only small insects to visit and contact the flowers. The bags were applied to inflorescences that had flower buds but no opened flowers. For control inflorescences, all insects had access to flowers, regardless of body size. At each study site, 6 and 12 inflorescences from different plants were selected, respectively, for the bagging treatment and control, consisting of equal numbers of thrum and pin inflorescences. The treatment and control inflorescences were marked with vinyl tape on September 3–5, when buds were about to open. These inflorescences were collected on October 7–10, when seeds ripened. As buckwheat produces one seed per flower, the number of seeds and remnant wilted flowers were counted and the seed set was defined as (number of seeds)/(total number of flowers). The difference in seed set between the treatment and control groups represents the contribution of large insects, as wind pollination is rare^43^. Therefore, the small insect contribution was defined as the average seed set from bagged inflorescences at each site. The large insect contribution was calculated by subtracting the average small insect contribution from the average seed set of controls at each site.

### Analysis

#### Insects analyzed

We categorized insects into seven taxonomic groups: ants, bee, scarabaeid beetle, butterfly, non-syrphid fly, syrphid fly, and wasp. These insects were considered potential pollinators for buckwheat^53,54^, although no direct evidence for pollination contribution is provided. All of these were used for size-based analysis (see “Response to weather and spatial structures”), but bees were not included in the taxonomic-level analysis because they were rarely observed throughout the surveys and their visitation records was not sufficient for statistical analysis. Other insects, including lady beetles, stink bugs, and grasshoppers, as well as spiders, were not included in the analysis, because of their lower frequencies of observation and/or predatory nature.

#### Spatial and landscape variables

To check the spatial autocorrelation of insect visitation frequency across study sites, we tested the significance of global Moran’s I for each taxonomic group and body size group. As a result, none of the autocorrelations turned out to be significant (p > 0.05). This, however, does not mean there were no spatial structures. Spatial structures of study sites were represented by Moran’s eigenvector maps (MEM), which describe synthetic spatial patterns of study sites in a two-dimensional space. MEM is often used for multiple regression or canonical analysis to account for spatial dependence (or spatial filtering) and to infer large scale environmental factors affecting spatial gradients^55,56^. MEM axes are automatically extracted from large- to fine-scale patterns, and each spatial pattern is quantified by the eigenvalue of the MEM axis^56^. We constructed MEMs using the coordinate data for study sites, and Delaunay triangular and MEM scores were calculated using the R package adespatial^57^. Significant MEM scores (p < 0.05 for Moran’s *I*) were used in subsequent analyses.

We examined the relationship between selected MEM scores and land cover by a redundancy analysis (RDA). Land cover was categorized into five types; forest, buckwheat field, agricultural field (other than buckwheat), residential area, and others (areas that do not belong to any of the four types). The area of each land cover within a 200-m-radius buffer from the edges of each study site was calculated using QGIS 2.18.23. This buffer size was based on earlier studies that estimated spatial scale of insects other than *Apis* and *Bombus* that were rarely captured in our survey. Taki et al. examined insects visiting buckwheat in Japan, and found that the spatial scale that best explained the visitation frequency of non-bee insects was about 100 m from the field margins^54^. Other papers described 200–300 m as the appropriate buffer size for insects other than *Apis* and *Bombus*^26,58,59^. We therefore used 200 m buffer radius in our study, and buffers generated from fields did not overlap substantially. In addition to simple land cover area, the Shannon diversity index of land cover proportions, which represents the mosaic of surrounding land use, was also calculated for each site. RDA was performed using the R package vegan^60^.

#### Response to weather and spatial structures

We analyzed the responses of pollinators to weather conditions and spatial structures by two approaches. First, analyses were performed for each taxonomic group. Second, the difference in responses between small and large insects to weather and spatial variables was examined.

We estimated the response of each taxonomic group by GLM using Bayesian modeling, as Bayesian framework provides more robust parameter estimation for complex model structures. The visitation frequency of each insect taxon in each site (number of individuals summed over four quadrats in a site) was used as a response variable. The three weather variables (temperature, sunshine duration, and wind speed), time, and MEM scores were used as explanatory variables. Note that none of the weather variables were highly correlated (VIF < 1.38) and thus no serious collinearity problems. Time was included to consider potential trends in pollinator activity rates during the day. Time was quantified as the difference between the earliest observation starting time (8:32) and the starting time for each observation. In addition, the site ID was included in the model as a random variable, making it a hierarchical model with daily observations nested in sites. A negative binomial model was fitted to the visitation frequency data to cope with overdispersion, using the R package “brms”^61^. A non-informative prior distribution was used for all explanatory parameters in the model. Posterior samples and distributions were obtained by MCMC sampling with 7000 iterations and 3500 burn-in samples. The number of chains was 2 and adapt_delta was set to 0.99. Convergence was confirmed by checking if R-hat (a measure of parameter convergence) was below 1.1^62^ for all estimates. Prior to the analysis, all explanatory variables were scaled into average 0 and variance 1, to save computation time.

We estimated the responses of small and large insects by GLM using Bayesian modeling.

The visitation frequency of insects in each size category (small or large) was used as a response variable. Body size (small or large), MEM scores, time, and three weather variables as well as two-way interactions between body size and environmental conditions (three weather variables, MEM scores, and time) were included as explanatory variables. A negative binomial model was used with a non-informative prior distribution for all explanatory parameters. The number of iterations, burn-in samples, and chains as well as adapt_delta were the same as those used in the first Bayesian model. All analyses were performed using R^63^.

#### Insect visitation and seed set

To confirm the linkage between insect visitation and pollination services, we analyzed the relationship between insect visitation frequency and seed set (no. of flowers that had produced seeds) by a simple regression, i.e., GLM with a Gaussian error distribution. The average seed set of controls at each site was included as the response variable, and insect visitation frequency averaged over all days at each site was used as the explanatory variable. Since the relationship between seed set and insect visitation frequency appeared non-linear, showing a decelerated increase with insect visitation frequency, log-transformation was performed for insect visitation frequency.

In addition, to understand the spatial pattern in the relative contributions of small and large insects to seed set, we conducted a multiple regression analysis separately for small and large insects, as in the above GLM model. The seed set from small insects or from large insects was used as the response variable, and four MEM scores were used as explanatory variables. It was not possible to test how the relative contributions of small and large insects vary with weather conditions because seed set was derived from all flowers in a season, rather than from flowers that bloomed on a particular day or time. All analyses were performed using R^63^.

## Results

### Observed insects

We recorded 6370 insect visitors in total, across sites and days. In terms of insect group composition, ants visited the flowers most frequently (2502 individuals), followed by scarabaeid beetles (1596), syrphid flies (697), non-syrphid flies (609), butterflies (259), wasps (219), and others (426). Only 62 bees were recorded. Small insects accounted for 3974 individuals and large insects accounted for the remaining 2396 individuals. Scarabaeid beetles (1596) accounted for more than half of large insects, whereas ants (2502) were most abundant small insects (Table 1). All butterflies were categorized as large, and the majority of syrphid and non-syrphid flies were categorized as small.

**Table 1:** Taxonomic composition of large and small insects observed visiting buckwheat flowers combined at all sites (14 buckwheat fields).

Large insects		Small insects	
Taxon	Abundance	Taxon	Abundance
Scarabaeid beetle	1596	Ant	2502
Butterfly	259	Hoverfly	538
Syrphid fly	159	Non-syrphid fly	463
Non-syrphid fly	146	Others	313
Others	113	Wasp	115
Wasp	104	Bee	43
Bee	19		
Total	2396	Total	3974

### Spatial structure of study sites

Four MEM axes (referred to as MEM1–4 hereinafter) were significant according to Moran’s *I*. The MEM scores for the study sites, representing different spatial structures defined by the four axes, are shown in Supplementary Fig. S2.

**Figure 1:**
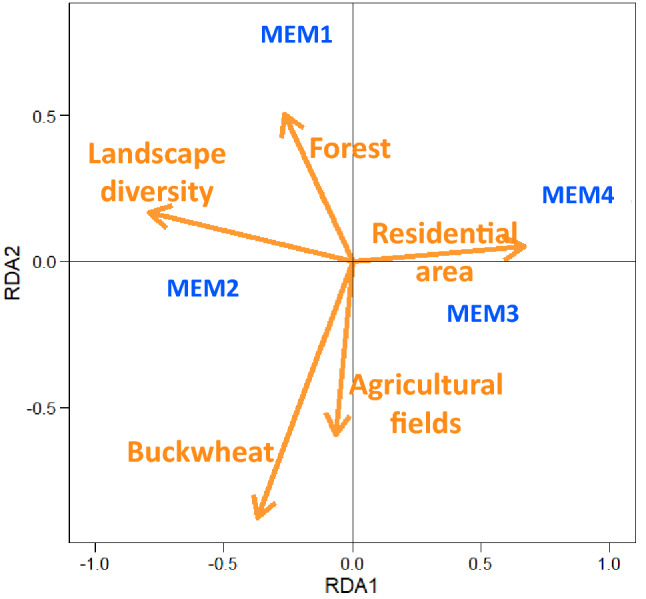
Results of RDA relating four spatial variables (MEMs: Moran’s Eigenvector Map) to five landcover variables, as represented by the two RDA axes. MEM scores were used as response variables and a set of landcover variables were used as explanatory variables (area proportions of 5 land cover types and landscape diversity (Shannon diversity index)). Arrows represent the vector of land cover variables, and the plotted texts (MEM1 ~ 4) indicate the locations of MEMs.

The results of the RDA linking MEM and land cover surrounding study sites are described in Fig. 1. Although the axes did not clearly represent land cover patterns, some general trends were observed. MEM1 was positively associated with forest. MEM2 was positively associated with the Shannon diversity index and negatively associated with residential area. MEM3 and 4 were positively associated with residential area.

### Response to weather and spatial variables

The initial analyses based on taxonomic groups showed that responses to weather conditions and MEM scores varied among taxonomic groups. R-hat was below 1.1 for all estimates, indicating the convergence of estimated parameters.

**Figure 2:**
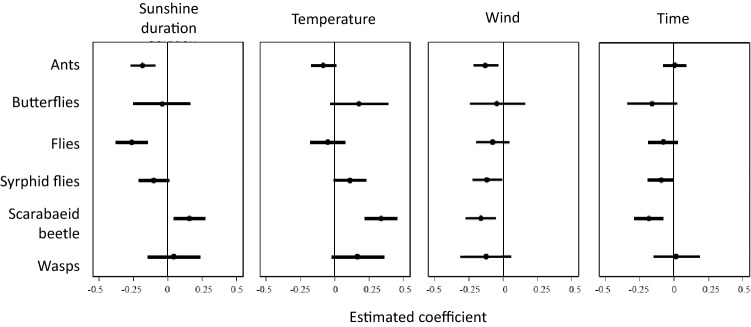
Results of GLM using Baysean analysis showing how weather variables and time infuence abundance of various insects visiting backwheat flowers. Lines indicate 95% credible intervals of the estimated coefficients of variables, and dots are the means of the coefficients. Note that positive coefficients represent positive responses of insects to weather and time variables. See Supplementary Fig. S3 for graphic representation of insect abundance vs independent variables.

With respect to weather variables, sunshine duration had a positive effect on the visitation frequency of scarabaeid beetles but negative effects on ants, non-syrphid flies, and syrphid flies (Fig. 2). The visitation frequencies of butterflies and wasps were not affected by changes in sunshine duration (Fig. 2). Ants were less active with higher temperatures, whereas other groups (except non-syrphid flies) tended to be higher in their activities as the temperature increased (Fig. 2). Wind speed was generally associated with a lower visitation frequency across taxonomic groups, although butterflies, non-syrphid flies, and wasps were more resilient to changes in wind speed (Fig. 2). Butterflies, non-syrphid flies, hoverflies, and scarabaeid beetles were less abundant as time progressed from early morning to early afternoon, whereas ants and wasps were stable, regardless of time of day (Fig. 2). The response curves of each taxon to weather variables are shown in Supplementary Fig. S3.

**Figure 3:**
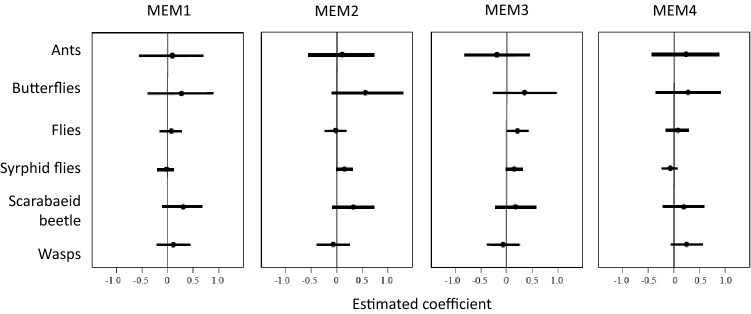
Results of GLM using Baysean analysis showing how spatial variables (MEM axes) infuence abundance of various insects visiting backwheat flowers. Lines indicate 95% credible intervals of the estimated coefficients of variables, and dots are the means of the coefficients. Note that positive coefficients represent positive responses of insects to MEM variables.

Concerning spatial structures, MEM1 showed no clear associations with any insects; however, it had a weak positive association with scarabaeid beetles (Fig. 3). This indicates that these species may be more abundant in more heavily forested areas, as MEM1 lies in the direction of "Forest” vector (Fig. 1). MEM2 was positively associated with butterflies, hoverflies, and scarabaeid beetles (Fig. 3), indicating that these groups are more abundant when the surrounding land use is diverse (Fig. 1). MEM3 was positively related to flower visitation frequencies of non-syrphid flies and hoverflies (Fig. 3); thus, these taxa may increase in sites with more residential areas (Fig. 1).

**Figure 4:**
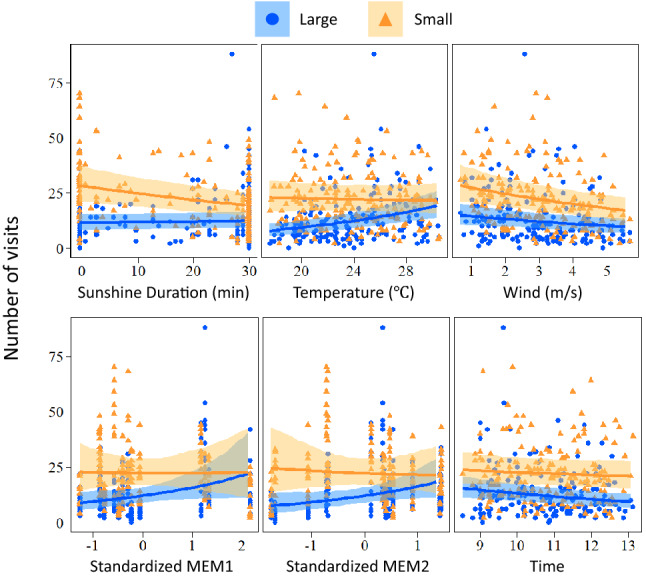
Relationship between weather variable or spatial variable and insect abundance for large and small insects, which was estimated by GLM. Error bands represent 95 credible intervals. Each dot represents the number of individuals observed at each census. MEM 3 and MEM 4 are not shown due to their insignificant effects. Note that MEM variables are standardized.

The second analyses based on body size showed that size, temperature, wind, time, and MEM2 have significant effects on the overall visitation frequency of pollinators (Table 2). R-hat values were less than 1.1 for all estimates, again indicating parameter convergence. Several interaction terms between body size and weather or spatial variables were significant based on the 95% credible intervals, including body size × temperature, body size × solar radiation length, body size × MEM1, and body size × MEM2 (Table 2). Small insects’ activity increased as the sunshine duration decreased but were not related to temperature, MEM1, or MEM2 (Fig. 4). In contrast, large insects were more active with higher temperatures and higher values of MEM1 and MEM2 but showed no response to sunshine duration (Fig. 4). Overall, large insects visited flowers more actively in fine or hot weather and became more abundant when the surrounding landscape was forested or mosaic structure. In contrast, small insects were more active when the weather conditions were rather inclement and were not strongly influenced by spatial variables. Wind speed had a negative influence on both large and small insects.

**Table 2:** Results of GLM using Bayesian analysis, showing the effects of insect body size (large or small), three weather variables, and four spatial variables on the abundance of insects visiting buckwheat flowers.

Variable	Estimate	− 95%CL	+ 95%CL
Intercept	2.40	2.11	2.68
**Size**	**0.61**	**0.52**	**0.71**
Sunshine duration	0.04	− 0.05	0.13
**Temperature**	**0.23**	**0.14**	**0.32**
**Wind speed**	**− 0.11**	**− 0.20**	**− 0.03**
Time	− 0.13	− 0.20	− 0.05
MEM1	0.26	− 0.03	0.54
**MEM2**	**0.29**	**0.01**	**0.57**
MEM3	0.09	− 0.19	0.39
MEM4	0.12	− 0.16	0.41
**Size × sunshine duration**	**− 0.20**	**− 0.32**	**− 0.09**
**Size × temperature**	**− 0.25**	**− 0.37**	**− 0.13**
Size × wind speed	− 0.02	− 0.13	0.09
Size × time	0.10	− 0.01	0.20
**Size × MEM1**	**− 0.27**	**− 0.37**	**− 0.17**
**Size × MEM2**	**− 0.33**	**− 0.44**	**− 0.23**
Size × MEM3	− 0.09	− 0.19	0.01
Size × MEM4	0.01	− 0.09	0.10

Spatial variables were represented by four MEM axes (Moran’s Eigenvector Map). The variables with bold lettering indicate their 95% credible interval do not overlap with zero.

### Insect visitation and seed set

**Figure 5:**
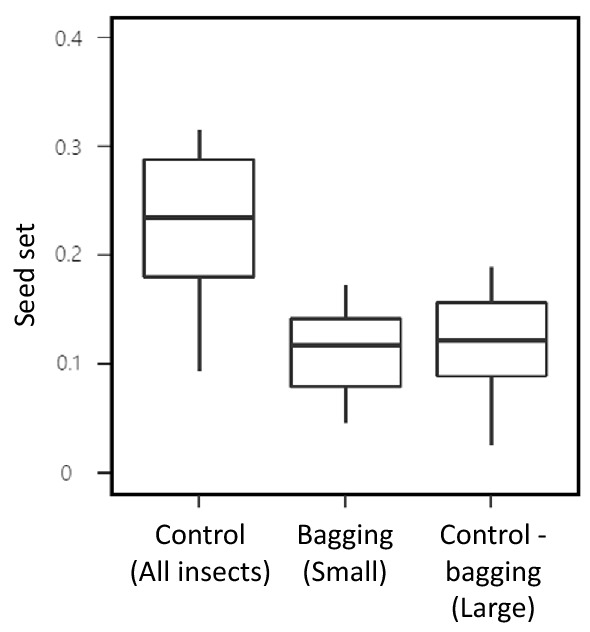
Seed sets of buckwheat (number of seeds/number of flowers) of control inflorescences (open to all insects), bagging inflorescences (only small insects had access), and the subtraction of bagging seed set from the control seed set. The three seed sets represent, respectively, the contributions of all insects, small insects, and large insects.

The seed set from control plants (exposed to natural pollinators) averaged over all sites was 0.24 (24% of the total flowers), and the small and large insects made nearly equal contributions to seed set of buckwheat (Fig. 5).

The seed set showed significant positive relationships with the visitation frequencies of the following insects visiting buckwheat field (Supporting information 4); ants (r = 0.631, p = 0.016), butterflies (r = 0.657, p = 0.025), scarabaeid beetles (r = 0.541, p = 0.046), and wasps (r = 0.536, p = 0.048). With regard to body size, seed set had a positive association with both large insects (*r* = 0.629, p = 0.016) and small insects (*r* = 0.533, p = 0.050) (Supplementary Fig. S4). The partial correlation between seed set and insect abundance was 0.562 for large insects and 0.436 for small insects, suggesting both insect groups made a net contribution to seed production. The seed set had also a positive association with the total number of visitations by all insect groups of observed insects (*r* = 0.671, p = 0.009) (Supplementary Fig. S4).

A multiple regression analysis showed that only MEM2 was positively correlated with the contribution of large insects to seed set (Table 3). This indicates that the contribution of large insects to pollination increases with increasing diversity of surrounding land cover types, with fewer residential areas, as shown in Fig. 1. However, none of the MEM scores were significantly related to the contribution of small insects to seed set (Table 3), indicating contribution of small insects to pollination is insensitive to surrounding land cover.

**Table 3:** Results of multiple regression analysis relating seed set of buckwheat to spatial variables represented by four MEM axes (Moran’s Eigenvector Map).

	Large insects		Small insects	
	*t*	*p*	*t*	*p*
MEM1	− 0.347	0.737	1.215	0.255
**MEM2**	2.813	**0.020**	0.297	0.773
MEM3	0.005	0.996	0.323	0.754
MEM4	0.968	0.358	0.323	0.222

The analysis was conducted separately for seed set contribution of large and small insects. The variable with a bold letter indicates statistical significance.

## Discussion

We detected differences in responses to changes in weather conditions among insect groups visiting buckwheat flowers. In particular, the responses of beetles, butterflies, and wasps differed from those of ants and non-syrphid flies; the former group was more active in sunny and/or warm conditions, whereas the latter taxa showed the opposite pattern (Fig. 2). Responses to weather conditions varied with insect body size as well; large insects increase their activity in high temperatures, whereas small insects increase their activity in cloudy weather conditions (Fig. 4). This contrasting pattern agrees with a previous study showing that insect body size is an important determinant of niche positions at ambient temperatures^10^. Moreover, small insect contribution to the seed set was tantamount to the contribution by large insects (Fig. 5). Thus, temporal complementarity among insect taxa or functional groups is likely to stabilize pollination service of buckwheat in variable weather conditions.

Many studies have reported diversity of pollinator responses to weather conditions among bee species visiting crops. Honeybees are generally active in high temperatures, while wild bee such as *Bombus*, *Osmia*, and *Anrena* are more active under low temperature or windy weather^7,10,34,48^. Moreover, dipterans have different responses to temperatures and sunshine duration than those of honeybees and wild bees, with dipterans being active even under inclement weather conditions^9,34,48^. To our knowledge, our study is the first to show diverse responses to weather conditions in pollinator communities visiting a single crop species, including Hymenoptera (bees, wasps, and ants), Diptera, Lepidoptera, and Coleoptera (but see Kühsel and Blüthgen^10^ for wild flowers, rather than a crop species). Most earlier studies focused on crops that are highly dependent on bee pollination, such as blueberry, strawberry, watermelon, pumpkin, and almond^7,16,28,35^. As buckwheat was visited by a variety of insect taxa in addition to bees, response diversity might have been more clearly demonstrated in this system. Our study classified insects with coarse taxonomic levels, because of visual observation rather than collecting them. With a lower taxonomic classification, such as family or genus levels, more diverse response patterns of insects would likely have been found.

Some earlier studies have shown that honeybees and bumblebees are major buckwheat pollinators^33,64^, similar to their contribution to other well-studied crops. It is therefore surprising that only a few bees were observed in our study area. This small number of bee visits was also confirmed in a former study conducted in this region^65^. It is worth noting that, despite low bee visitations, seed set in our study fields was larger than that reported in a previously study (ca. 25% vs. 20%) showing frequent bee visitations^43^. This suggests that diverse insects other than bees should have overcompensated the deficit of bee pollination services.

Thus, the insect diversity apparently contributed to the pollination of buckwheat and seed production in our study area. Of particular note is that ant visitation frequency was positively correlated with seed set, and they accounted for a substantial portion of small insects. Moreover, ant visitation frequency had lower correlations with the visitations of other insects (Supplementary Fig. S5). Ants are major pollinators of some wild plants^66,67^, but ant pollination of crop plants has not been reported, except for mangoes in the tropics^68^. However, our recent experimental study revealed a significant contribution to buckwheat pollination by ants^69^. Ants do not move long distances, and pollination may be limited to a small spatial range^68^. However, given that buckwheat is cultivated densely and two types of distylous flowers are close by, pollen transfer by ants is likely to be prevalent.

Although it is not possible to quantify the exact degree to which response diversity to weather conditions affects the pollination service of buckwheat, its contribution is likely to be significant because the contributions of large and small insects to seed set were nearly equivalent, as evaluated by the bagging field experiment. Small insects were mainly ants and dipterans, whereas large insects were mainly beetles and butterflies, and these groups appear to have independent contributions to seed production in buckwheat. In addition, given that there were significant statistical interactions between the body size of insects visiting flowers and weather conditions (both temperature and sunshine duration; Table 2), response diversity is likely to have an important role in the stabilization of seed set of buckwheat. Further studies are required to link visitation frequency with seed set for various insect groups. As a caveat, bags could be visible to some flying insects and prevented access to flowers even for small insects, which might have underestimated contribution of small insects. This does not alter the conclusion, however, that small insects had a significant contribution to seed set of buckwheat, as it was certain that only small insects were able to get access to flowers in the bagging treatment. We did not quantify the pollination by wind, but it is known to be negligible (less than 2% seed set)^70^, which does not undermine our conclusion.

It is noteworthy that we simultaneously estimated the response to three weather variables and time of day with a single statistical model, and the pure responses to weather variables were therefore extracted, without the confounding effect of time. In this analysis, temperature disproportionately affected large insects, whereas sunshine duration tended to affect small insects (Fig. 4). This implies that patterns of response diversity differ between environmental parameters. Different effects of weather variables on response diversity were also observed at the taxonomic level. Although the patterns of response diversity among taxonomic groups were qualitatively similar for temperature and sunshine duration, the response intensities, as measured by the estimated parameter values, were different. For instance, the activity of syrphid flies increased under high temperatures, but decreased under long sunshine durations (Fig. 2). Additionally, butterflies and wasps were more active under higher temperatures, but a similar trend was not found for sunshine duration (Fig. 2). In general, temperature and sunshine duration are correlated; however, this correlation appears to be relatively weak when warm air and cold air are frequently exchanged, such as in the spring and autumn. Moreover, temperature is more stable within a given day compared with sunshine duration, which can change over short time periods; accordingly, separate effects of the two weather variables on insect visitation may have been detected.

These findings have important implications for our general understanding of response diversity to environmental changes other than weather variables. Earlier studies have focused on response diversity along a single environmental gradient. However, multiple environmental changes (e.g., increased CO_2_, nitrogen, and grazing intensity) affect particular ecosystem functions^71^. This indicates that a greater species richness is required to maintain ecosystem functioning in comparison with that required for a single environmental change^71^. Moreover, climate change could be accompanied by changes in multiple environmental parameters, such as temperature and precipitation, and individual species may have different sensitivities to these changes^39,72^. In addition, other variables, including sunshine duration and humidity, are also expected to change^73,74^. In this study, we found that the response to a specific weather variable was diverse among taxa and that such a diverse response itself also differed in different weather variables. We therefore suggest that response diversity at multiple levels deserves more attention in future studies of the biodiversity–ecosystem service relationship.

In addition to response diversity to weather, responses to spatial structures also differed between large and small pollinator insects; large insects tended to be more abundant in fields with more surrounding forest (MEM1) and landscape diversity (MEM2), whereas small insects did not exhibit such patterns (Figs. 1, 4). This suggests that small insects are less sensitive than large insects to habitat alterations, which may compensate for a decrease in large insects visiting buckwheat fields. This is consistent with a previous study showing that smaller insects are less sensitive to land-use intensification in agricultural landscapes^10,25,30,35^. As a caveat, the buckwheat fields examined in this study were distributed on a narrow spatial scale, and landscape-level replication was not possible. Instead, we used MEM variables to represent spatial structures. MEM variables are purely dependent on the positions of the buckwheat fields in a 2-dimensional space. Given that the spatial structures are not based on particular land cover types, their interpretation with respect to environmental gradients is not straightforward. In addition, the observed effects of landscape structures may simply reflect chance events occurring at a local scale, irrespective of land cover types. Despite these limitations, the different responses of insect groups with different body sizes to spatial structures provides evidence for spatial complementation, even if such a difference arose by chance. It should be noted that the contribution of large insects to seed set was positively related to MEM2 associated with landscape diversity (Fig. 1, Table 2), whereas that of small insects was not. This suggests that the spatially different visitation rates of large and small insects were translated to pollination services of buckwheat, and that small insects may sustain crop production under habitat alterations, as suggested previously^35,41^. Further studies on buckwheat in other regions, as wells as on other crop species, are required to generalize the importance of response diversity of pollinators for ensuring resilience to environmental changes.

In conclusion, we detected temporal and spatial response diversity of various insects visiting buckwheat, which contributed to the seed production in buckwheat, as suggested partially by a bagging experiment. A particularly important finding is that response diversity per se is also diverse in relation to different weather variables. There are many temporal dimensions, including yearly, seasonal, and daily changes, and long-term change affects population-level change while short-term change induces behavioral change. All of these should be considered when attempting more precise future predictions of pollination services under climate change. The final point to note is that, due to the limited number of study fields, we could not test the interactive effects of spatial and temporal factors or the interactive effects of weather factors (temperature and sunshine). Given that interactive effects could mitigate the detrimental effect on pollinators caused by single factor^39,40^, further studies should investigate how interactive effects of multiple environmental variables stabilize or destabilize pollination services.

## Supplementary Information

Supplementary Figures.

## Data Availability

The datasets generated during and/or analyzed during the current study are available from the corresponding author on reasonable request.
