# Supplementary material for: Diverse flower-visiting responses among pollinators to multiple weather variables in buckwheat pollination
Source: Sci Rep. 2023 Feb 22;13:3099. doi: 10.1038/s41598-023-29977-z (PMC9946946; doi:10.1038/s41598-023-29977-z)

**Supplementary Figure S1**


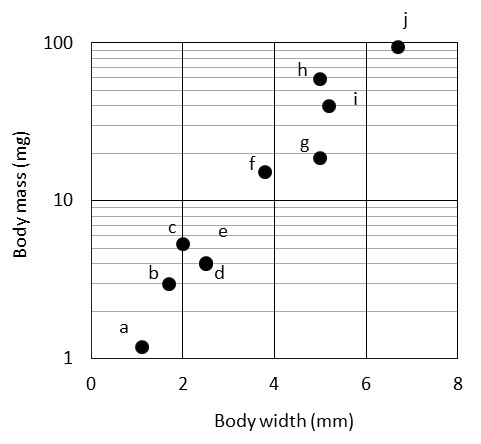


Figure S1. Relationship between body width of major insects visiting buckwheat flowers and their estimated body mass.

a: *Formica japonica* (Hymenoptera), b: *Sphaerophoria macrogaster* (Diptera), c: Halictidae (Hymenoptera) d: *Stomorhina obsolete* (Diptera) e: Ichneumonidae (Hymenoptera) f: Calliphoridae (Diptera), g: *Eristalis tenax* (Diptera), h: *Parnara guttata* (Lepidoptera), i: *Polistes chinensis* (Hymenoptera), j: *Mimela splendens* (Coleoptera)

A preliminary sampling of insects visiting buckwheat flowers were conducted in the study area in September of 2018. The body width and length of major species were measured. For major insects in different taxa and with different body size, body mass (mg, dry weight) was estimated from the body length, using allometric equations for insects in different families (Rogers et al. 1977). Note that Halictidae varied in body size but most samples were around 2mm body width and those >3mm were only 3% to the total number.

References

Rogers et al. 1977. Length-weight relationships of shrub-steppe invertebrates. Ann Entomol Soc Am 70: 51–53.

**Supplementary Figure S2**

Locations of the buckwheat fields studied (denoted by squares), as represented by minimum spanning tree estimated by Moran’s Eigenvector Map (MEM). White and black squares indicate negative and positive MEM scores, respectively, and the square sizes are proportional to score values. The longest link was determined by an appropriate distance threshold (see Legendre and Legendre (2012) for details).


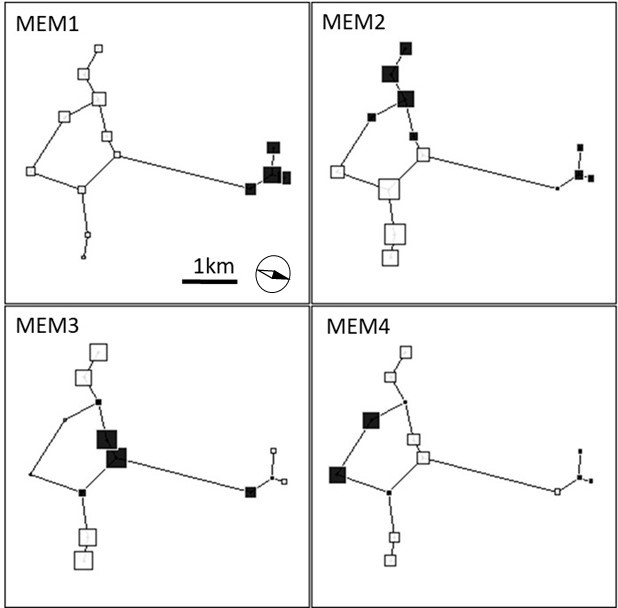


**Supplementary Figure S3**

Relationship between weather variables and insect abundance (Y-axis) for various taxa, which was estimated by generalized linear model. Error bands represent 95 credible intervals.

**
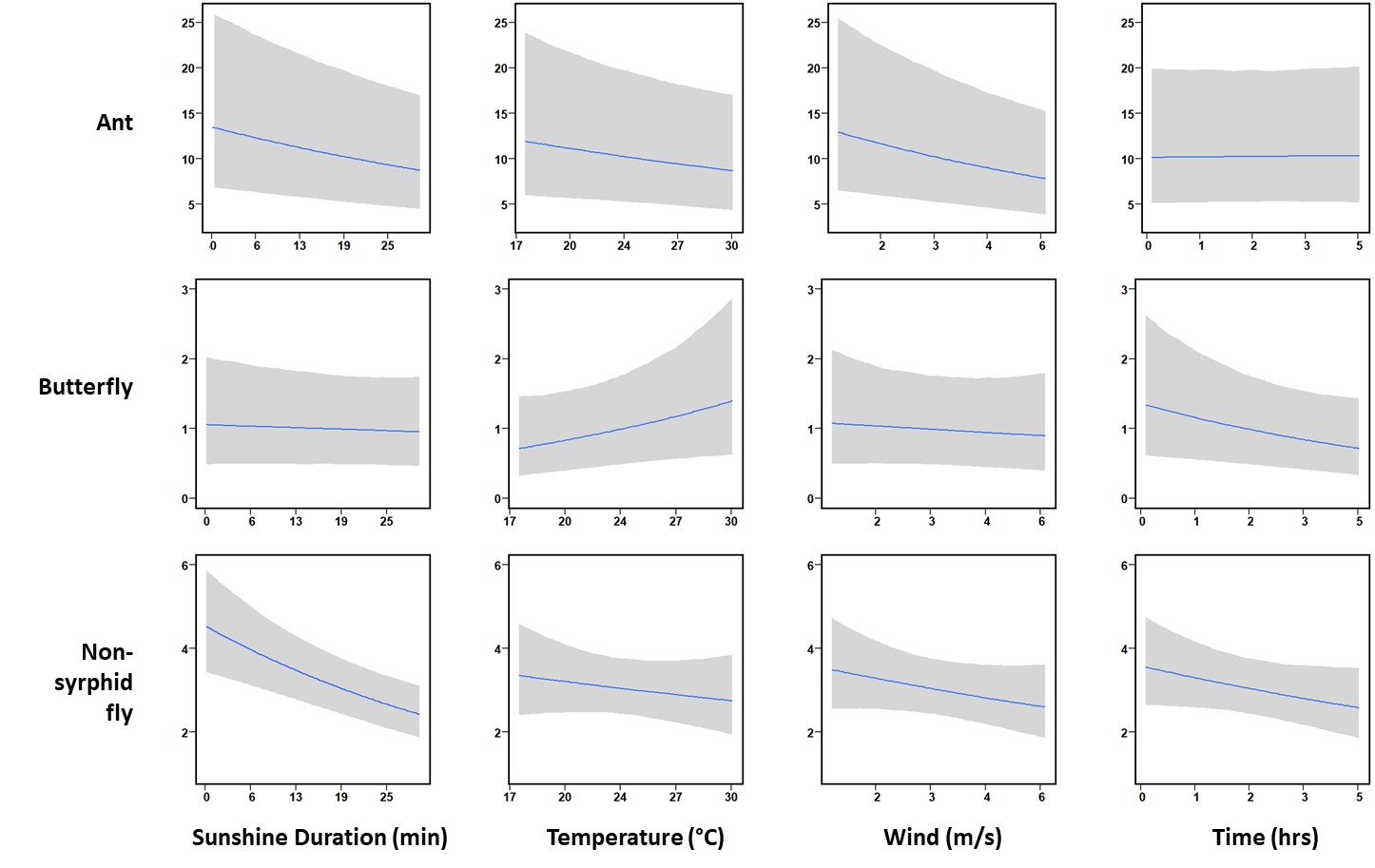
**

**Supplementary Figure S3 (continued)**


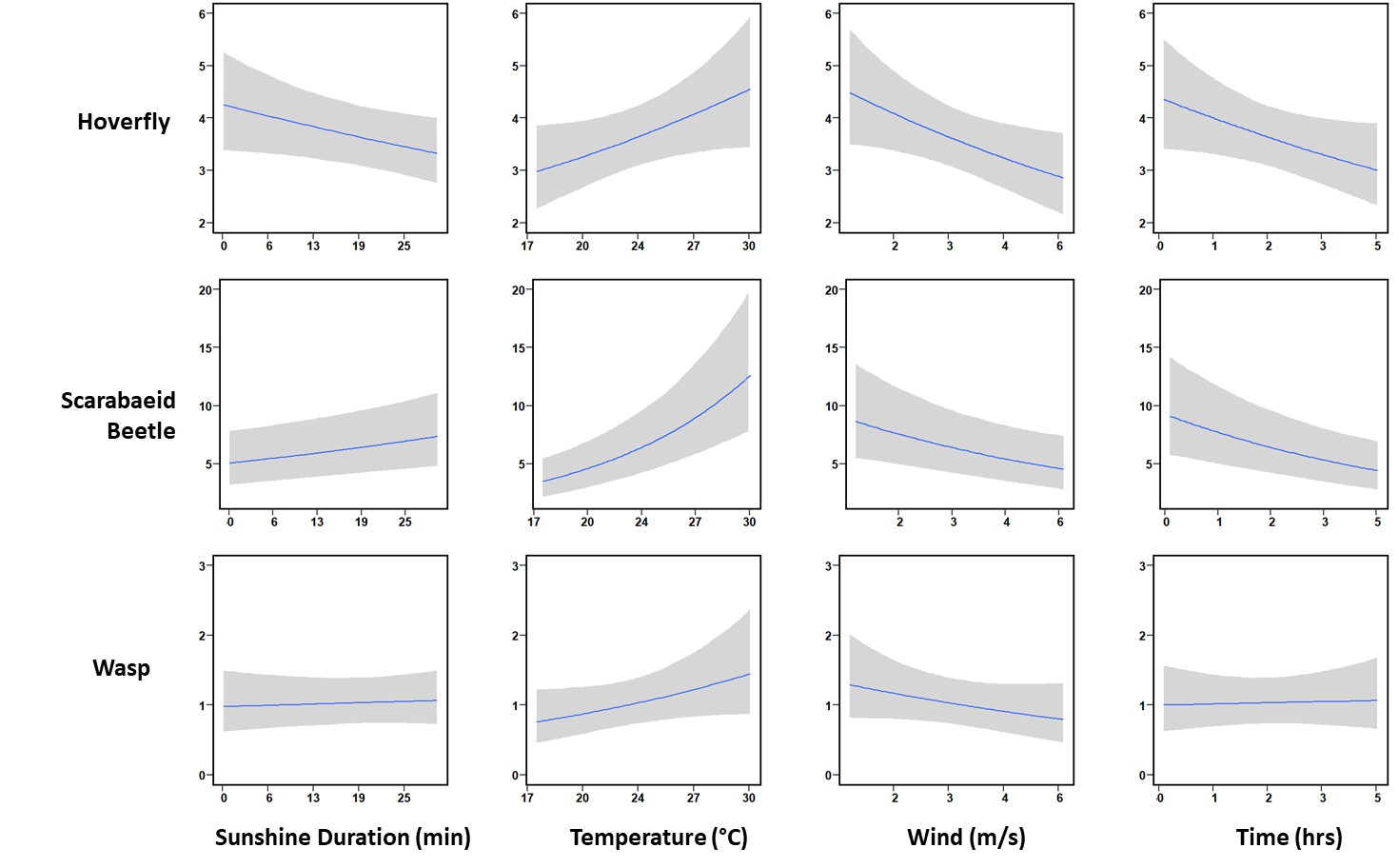


**Supplementary Figure S4**


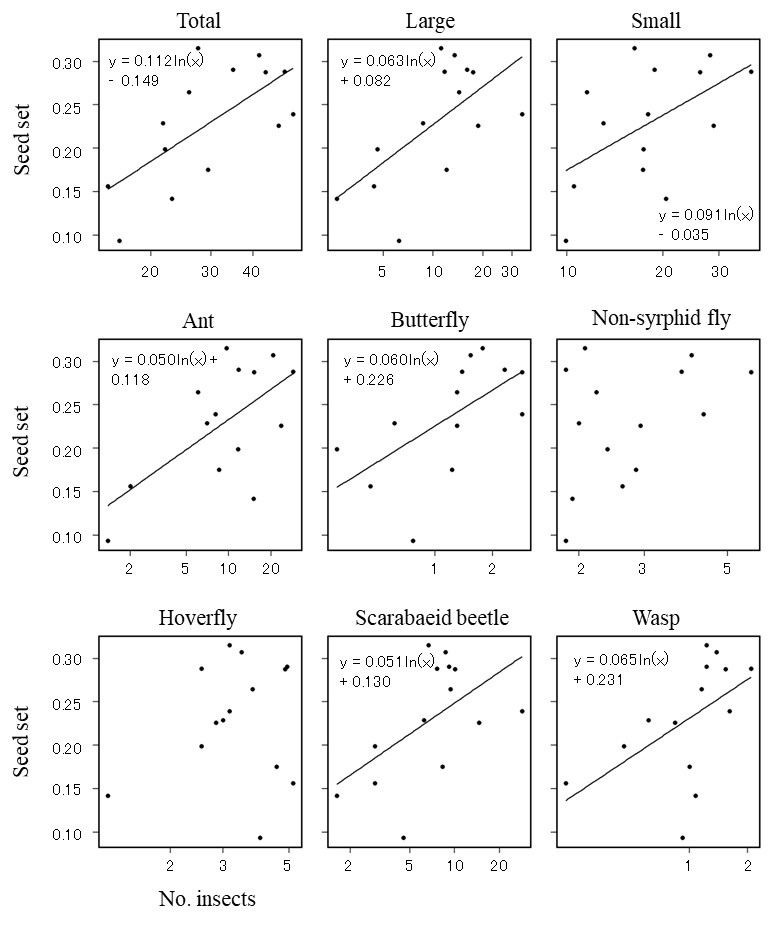


Figure S4. Relationship between mean seed set of buckwheat (no. of seeds/no. of flowers) in buckwheat fields and number of insects visiting there. Each plot represents the value in each study site, and regression lines have been drawn for significant associations.

**Supplementary Figure S5**

Correlation coefficient between the abundance of insects in different groups visiting buckwheat flowers at the field level.


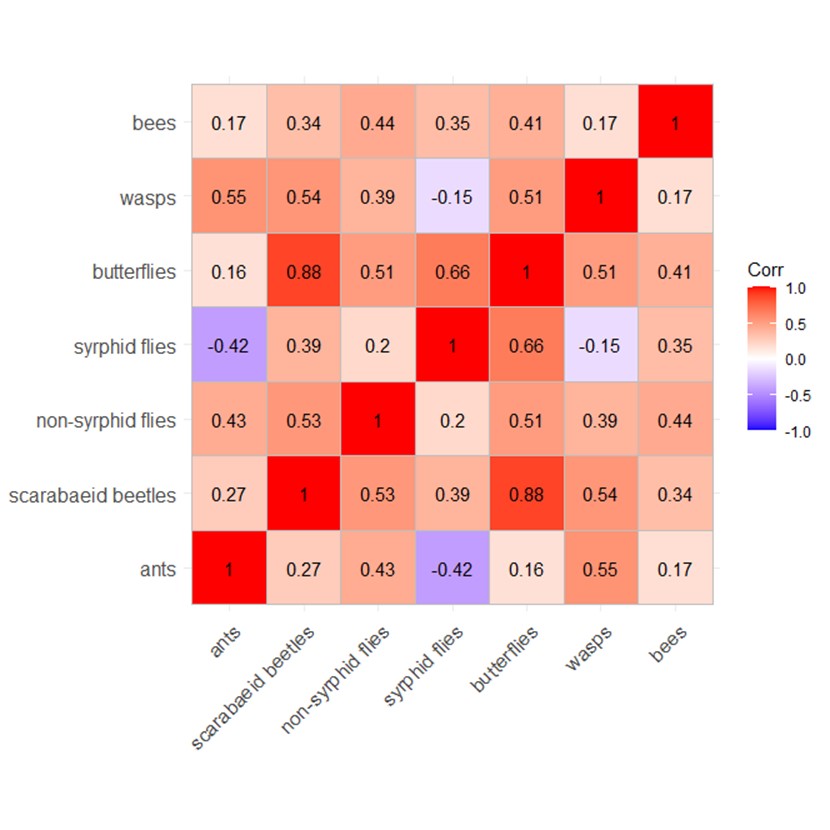

Supplement: Supplementary file 1 — Supplementary Figures. [file 41598_2023_29977_MOESM1_ESM.docx]
